# Orofaciodigital syndrome type II (Mohr syndrome): a case report

**DOI:** 10.1186/s12891-020-03825-x

**Published:** 2020-11-30

**Authors:** Bita Malekianzadeh, Fardis Vosoughi, Ramin Zargarbashi

**Affiliations:** 1 Anesthesiology Department, Tehran University of Medical Sciences, Tehran, Iran; 2 Department of Orthopaedic and Trauma Surgery, Shariati Hospital and School of Medicine, Tehran University of Medical Sciences, Tehran, Iran; 3 Department of Pediatric Orthopedy, Faculty of Medicine, Tehran University of Medical Sciences, Tehran, Iran; 4 Children’s Medical Center, Tehran University of Medical Sciences, Tehran, Iran

**Keywords:** Orofaciodigital syndrome, Mohr syndrome, Polydactyly, Central incisors

## Abstract

**Background:**

Orofacial digital syndrome is a rare genetic disorder with oral cavity, facial and digits anomalies. Orofacial digital syndrome type II, also called the “Mohr syndrome” is a very rare subtype that has been reported scarcely in Asia especially in Japanese patients.

**Case presentation:**

The case is an Iranian 5-year old girl who had been admitted for orthopedic surgery. She surprisingly had pre and postaxial polydactyly of all the four limbs concurrent with syndromic face and most of the features of Orofaciodigital syndrome type II.

**Conclusion:**

Mohr syndrome, anesthesia and surgical considerations are discussed in this case report. It is recommended to consider these considerations and the possibility of OFDS in every child with pre and postaxial polydactyly of the four limbs and to try to distinguish type II from other types of ODFS.

## Background

Polydactyly is a relatively common upper limb anomaly compared to other musculoskeletal congenital anomalies [1, 2]. Concurrent pre and postaxial polydactyly of multiple limbs with concomitant facial anomalies, on the other hand, is not so common. A child with multiple limb polydactyly and abnormal facial features should further be assessed to look for oral anomalies with suspicion of OFDS. Orofacial digital syndrome (OFDS) is a rare congenital disorder characterized by a range of clinical anomalies, such as malformations of the oral cavity including mouth, tongue, and teeth as well as developmental disorder of the face, head, nose, eyes, fingers, and toes. Most of these features are present at birth. Currently, at least 13 clinical types of OFDS have been identified which could be classified according to the existing anomalies [3–5]. Most of such children are born out of consanguineous marriages. OFDS can be inherited in form of autosomal dominant, autosomal recessive, X-linked recessive, or X-linked dominant. OFDS often has a recessive pattern, where both parents are mutation gene carriers. OFDS type I (Papillon-League and Psaume syndrome) is the most common type of OFDS with X-linked inheritance mode. The responsible gene for OFDS I could be diagnosed and confirmed through the genetic test. However, currently, there is no specific test for identifying the other types of OFDS. OFDS patients need medical care and some surgeries such as the reconstruction of cleft palate and limb deformities. OFDS type II or “Mohr syndrome” is a very rare disorder with an incidence of 1/1000000 worldwide. It was first described as a familial disease in 1941 by Mohr. OFDS type II is an autosomal recessive syndrome and the responsible gene has not been fully specified. Diagnosis is based on the clinical picture which has been rarely reported in Asian countries [6–9]. In this study, a five-year-old Iranian girl with Mohr syndrome who was admitted for orthopedic surgery is reported.

## Case presentation

**Fig. 1:**
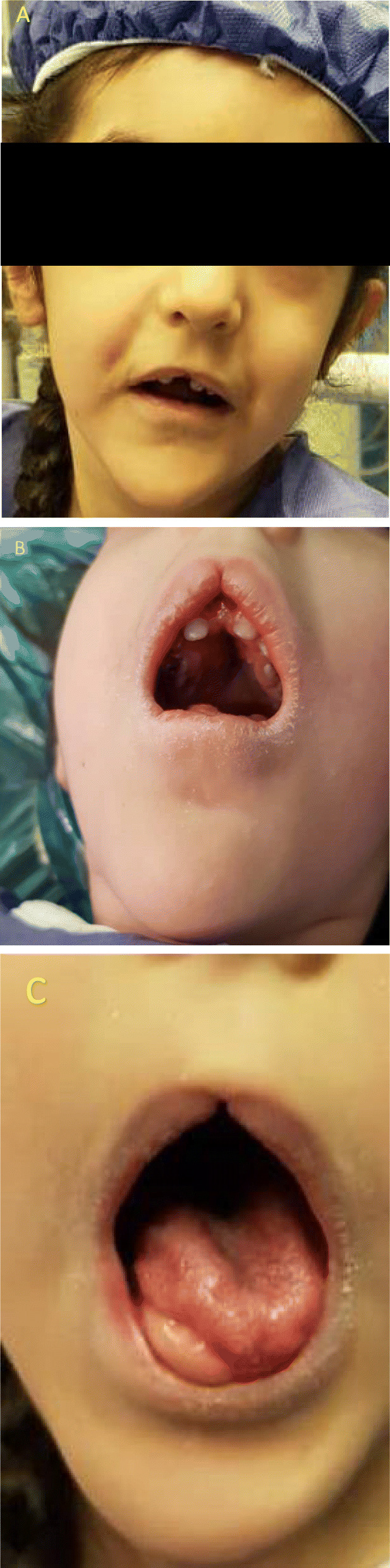
Orofacial manifestations of Mohr syndrome including the patient’s face (**a**), midline lip cleft (**b**), high-arch palate (**b**), absence of central incisors (**b**) and presence of tongue nodule (**c**)

**Fig. 2:**
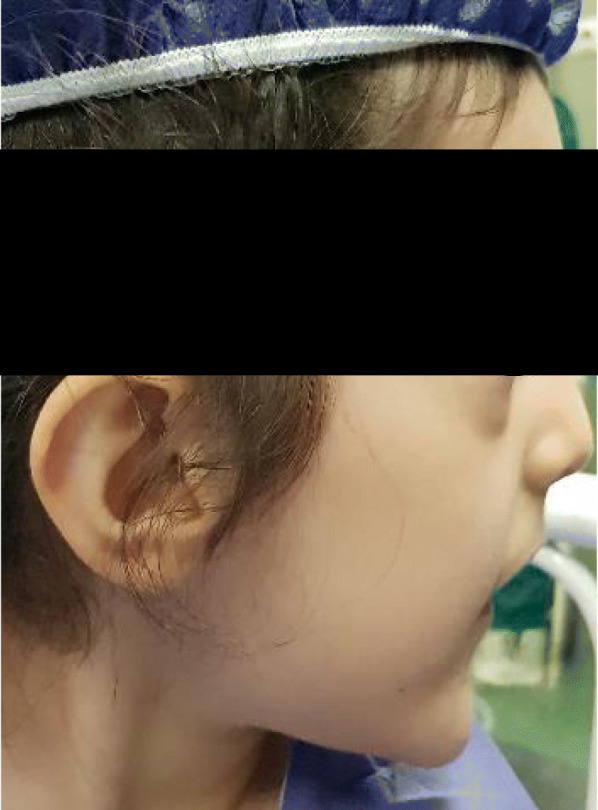
Low-set ear

**Fig. 3:**
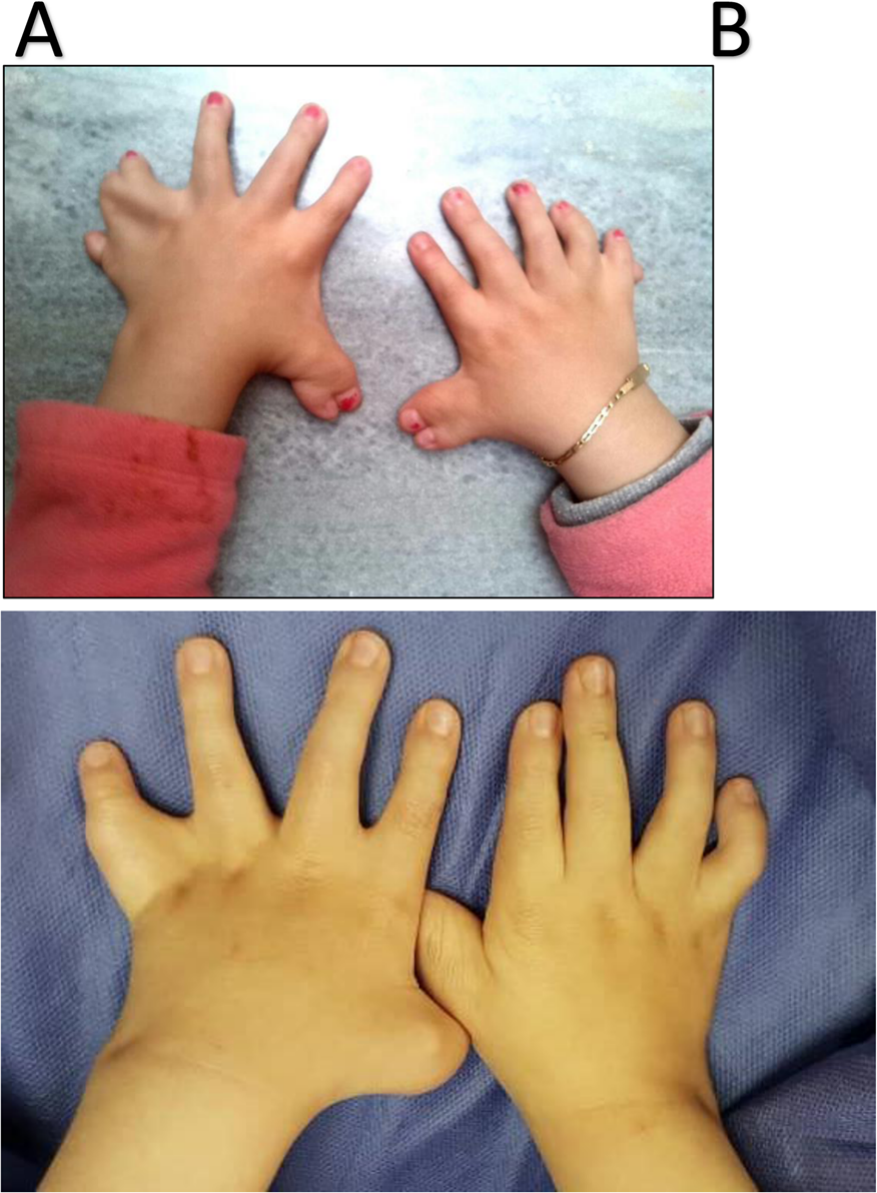
Pre and postaxial polydactyly of hands, before (**a**) and several years after (**b**) surgical correction

**Fig. 4:**
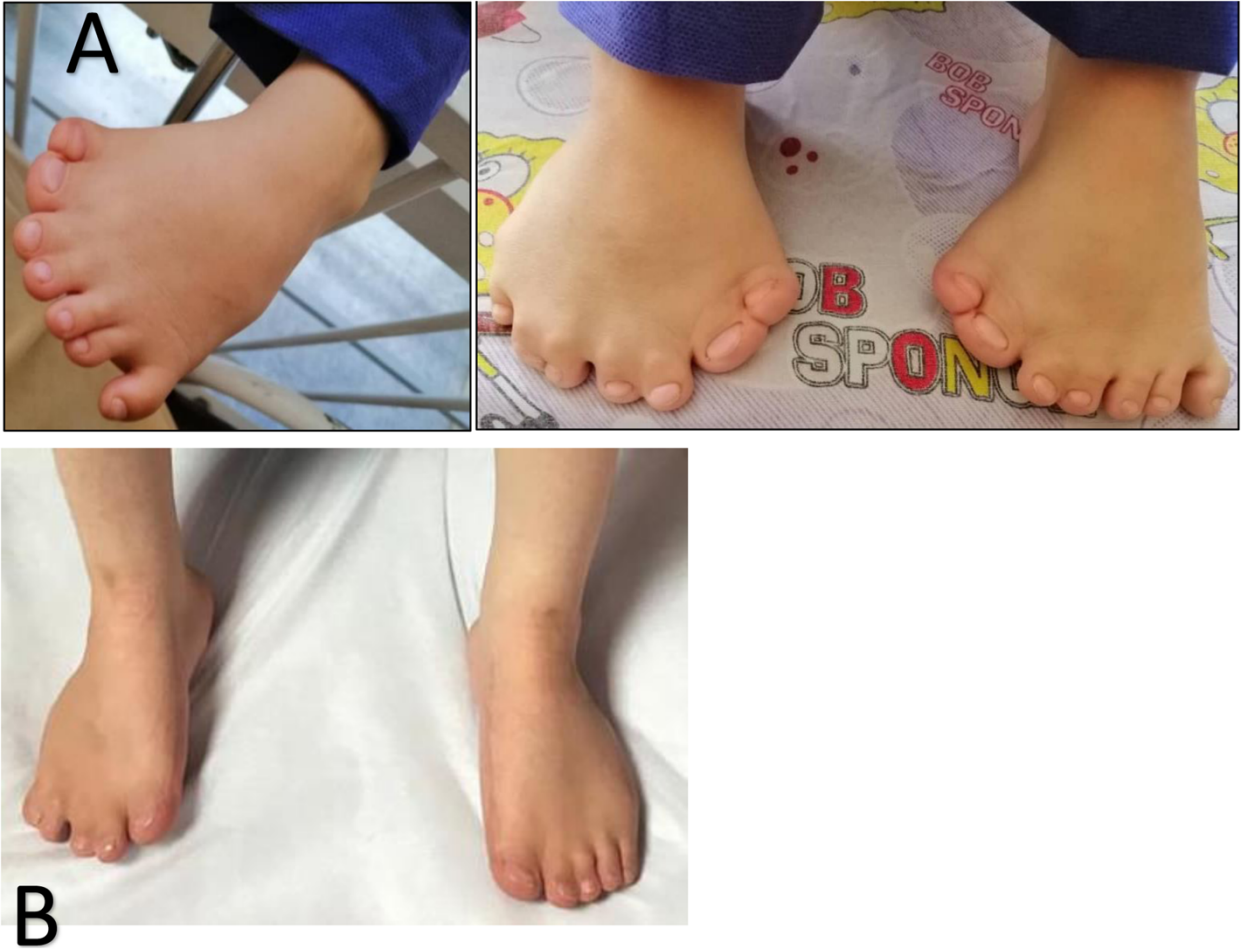
preaxial and postaxial polydactyly (mirror deformity) of the feet before (**a**) and one year after surgical correction (**b**) years old

**Fig. 5:**
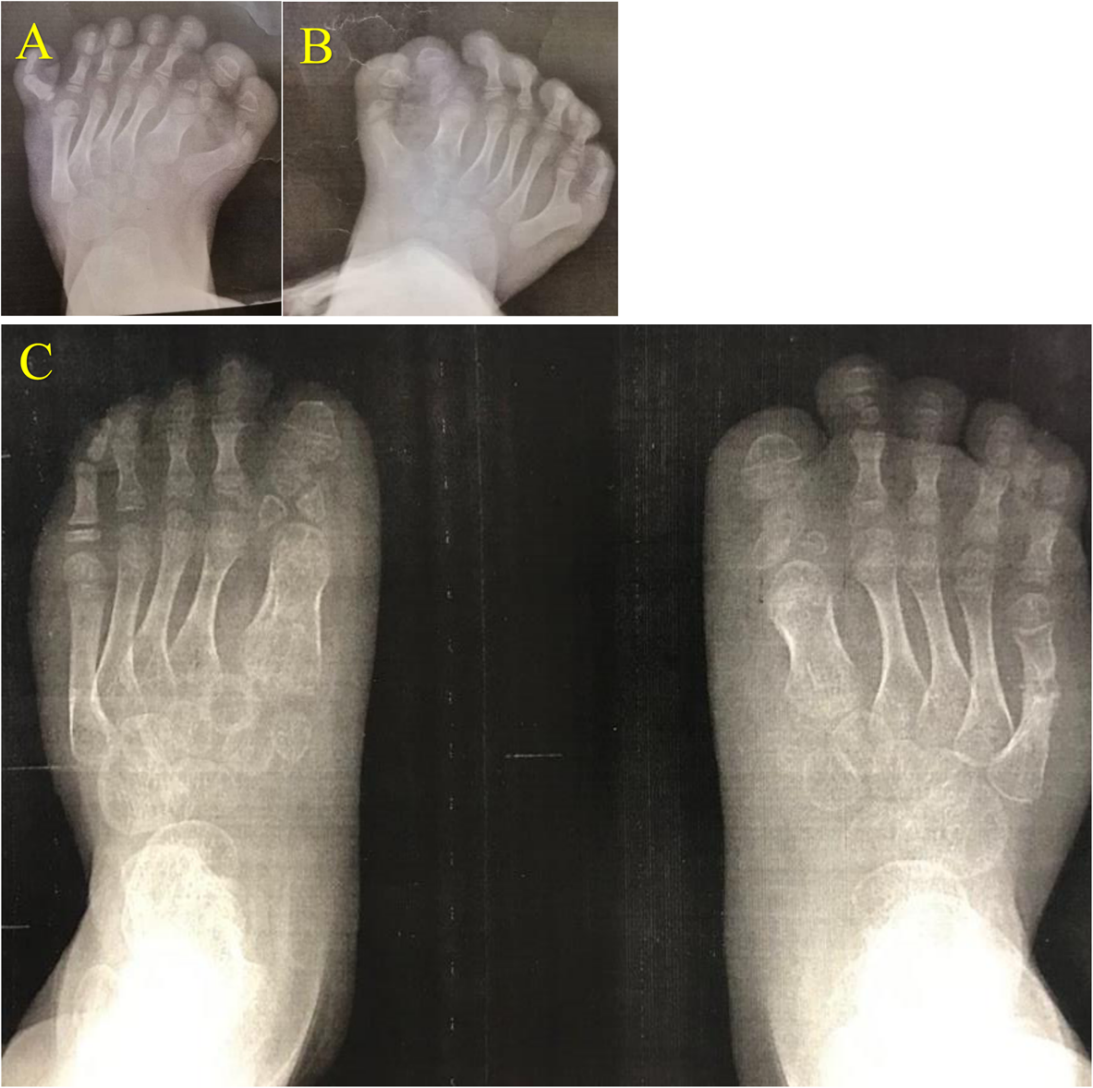
left (**a**) and right (**b**) foot anteroposterior radiographs of the patient showing bilateral polydactyly before and after (**c**) surgery

A five-year-old girl was admitted to a tertiary pediatric center in Tehran, Iran for the reconstruction of foot deformity. The child was born of consanguineous parents and full term at birth. Her parents seemed healthy with no prior medical history. She had undergone uncomplicated anesthesia for orthopedic hand surgery and lingual frenectomy three times. Her development had been delayed. Her mother mentioned that she suffered from obstructive sleep apnea. Lab data including Complete Blood Cell Count with leukocyte differentials, Blood Urea Nitrogen, Creatinine, PT, PTT, and INR levels were all within normal limits. Echocardiography showed no cardiac anomaly. She presented with typical features of OFDS type II (Fig. 1), which included mouth cavity and facial deformities, tongue nodules, missing central incisors, high arch palate, broad nose, midline lip cleft, hypertelorism, as well as low-set ears (Fig. 2), and limb deformities. Bilateral polydactyly of hands (Fig. 3) that was corrected in previous surgeries and bilateral pre and postaxial polydactyly of feet (Fig. 4) were among her list of anomalies. She was scheduled for surgery to correct the foot deformity (Fig. 5). After performing electrocardiography, pulse oximetry, and non-invasive monitoring of blood pressure, lidocaine 1.5 mg/kg and fentanyl 1 μg/kg were injected. Anesthesia induction was performed with propofol 2 mg/kg without any muscle relaxants. Direct laryngoscopy with Miller blade was done and uncuffed endotracheal tube was inserted. Laryngoscopic grade was II and dorsal port of vocal cords and bifida uvula were observed. Mirror foot deformity of right foot was corrected through a 94-min surgery. During the operation, the surgeon excised pre and postaxial rays in order to create a suitable foot for wearing shoes. Serial casting after the surgery was performed in order to stretch soft tissue contractures. At the end of surgery, the patient was extubated and after recovery, she was transferred to the surgical ward. She was stable without experiencing any complications during the anesthesia and recovery time.

## Discussion and conclusions

OFDS includes a wide variety of symptoms which make the diagnosis confusing for the pediatric orthopedic surgeon. The incidence of OFDS type I, the most common type of this syndrome, is 1/50000–1/250000 live birth. Mutation of chromosome X (22.3–22.2) is responsible for this disorder. This gene (Xp22) plays an important role in the development of body organs such as brain, face, limbs, and kidneys [10–12]. It encodes a protein localized in the centrosome and basal body of primary cilia. At present, OFDS appears to be a marked subgroup of ciliopathies with heterogeneity [13]. It is important to distinguish type I and II (Mohr syndrome). Type I is associated with cardiac and brain anomalies. Diagnosing Type II is mostly important because of the associated difficulties in the intubation process. This distinction also has important implications in genetic counseling [11]. If OFDS type I is suspected, genetic testing may confirm it. However, the gene associated with the other types is not yet recognized and the diagnosis is mainly clinical [14]. In this case study, we presented a patient with Mohr syndrome who underwent anesthesia for orthopedic surgery. The patient had some typical features of OFDS type II, which can be differentiated from type I and the other types, including high-arch palate, broad nose, hypertelorism, and tongue nodules. One characteristic which can help this differentiation is that the type I presents with milia or small noncancerous cysts on the face and ears [4, 9, 15].

Our patient was a sporadic case with oral, facial, and digital manifestations. The congenital absence of central incisors is a frequent feature of Mohr syndrome, whereas alar hypoplasia, alopecia, and milia are the main characteristics of OFDS type I. Renal involvement in the form of polycystic kidney disease is usually present in type I in contrast to type II. Distinguishing different types of OFDS is also important due to associated involvement of heart, kidney, and central nervous system, especially in type 1 [11, 16, 17]. In our case, these organs were not involved. Craniofacial anomaly and high arch palate in Mohr syndrome can be challenging for anesthesiologists. Difficulty in airway should be anticipated and discussed with the anesthesiologist beforehand. High-arch palate which is associated with some congenital syndromes, is a known cause of difficult laryngoscopy. It may also be difficult to place supraglottic devices such as laryngeal mask airway in these patients, so difficult airway equipment availability is recommended. However, in most of the reported cases, intubation was performed by an anesthesiologist without the use of these devices [18, 19]. Using a Miller blade, the Mallampati grade of our patient was 2 and head extension was normal. Mask ventilation was adequate and we did not encounter any difficulty during intubation.

Mohr syndrome is a very rare type of OFDS. Patients often need several surgical interventions from infancy to adulthood and the surgical team should be prepared for a difficult intubation. Video-assisted laryngoscopy might be safer in these patients.

Polydactyly of the 4 limbs (tetra polydactyly) is very rare, and so is simultaneous preaxial and postaxial polydactyly. We performed an extensive literature search and the only known associated entities were Ellis van Kreveld and OFD syndromes. Hence it is recommended to consider the possibility of OFDS in every child with pre and postaxial tetra polydactyly and then evaluate for associated brain, cardiac and maxillofacial anomalies and be prepared for a difficult intubation in these patients.

## Data Availability

Not Applicable.

## Abbreviations

OFDS: Orofacial digital syndrome
PT: Prothrombin Time
PTT: Partial thromboplastin Time
INR: International Normalized Ratio
